# Psychoeducation Programming for the New Caregiver: Mastering the First 100 Days

**DOI:** 10.1093/geroni/igaf122.4000

**Published:** 2025-12-31

**Authors:** Carolyn Clevenger, Karah Alexander, Molly Perkins, Fayron Epps, Kenneth Hepburn

**Affiliations:** Emory University, Atlanta, Georgia, United States; Emory University, Atlanta, Georgia, United States; Emory University School of Medicine, Atlanta, Georgia, United States; UT Health San Antonio, San Antonio, Texas, United States; Emory University, Atlanta, Georgia, United States

## Abstract

A dementia diagnosis marks a defining transition for individuals who must quickly assume the role of “caregiver.” While many programs exist for caregivers of persons living with dementia (PLWD), most are designed for those in the later stages of their conditions, when substantial cognitive losses have occurred and behavioral and psychological signs of dementia are manifesting themselves. This leaves a gap in support for caregivers at the time of initial diagnosis. As part of this two-year, two-phase project, we engaged a diverse group of experienced family caregivers to co-develop and prototype-test the Caregiver Bootcamp program, Caregiver Bootcamp is a synchronous online, interactive psychoeducation program aimed at building caregiving competence and confidence (mastery) among caregivers for persons recently diagnosed with dementia. We report on prototype testing outcomes and subsequent course revisions prior to the Stage Ib single-group clinical trial of the finalized program. Twenty-two caregivers participated in five live or recorded two-hour sessions over six-weeks. The course covered four key areas: processing the diagnosis and its implications (including self-care), understanding the basics of dementia conditions, taking the first legal and financial logistical steps, and building a support network. Participants were predominantly Black or African American (60.9%), female (87%), and were currently caring for a parent (55.6%). Based on feedback, program changes included shortening weekly sessions to 90 minutes, integrating video narratives from seasoned caregivers to illustrate real-world implementation of course recommendations, simplifying “academic presentation” of theoretical models, increasing interactivity, and expanding resource offerings to address various learning styles.

